# Parasocial interactions with media characters: the role of perceived and actual sociodemographic and psychological similarity

**DOI:** 10.3389/fpsyg.2023.1297687

**Published:** 2023-12-08

**Authors:** Michelle Möri, Andreas Fahr

**Affiliations:** Department of Communication and Media Research, University of Fribourg, Fribourg, Switzerland

**Keywords:** similarity, parasocial interaction, perceived similarity, actual similarity, experience sampling

## Abstract

**Introduction:**

Similarity between media character and viewer is an important predictor of parasocial interactions. Thereby, similarities are often limited to single characteristics or to the similarities viewers perceive between themselves and characters. This article expands the existing literature in two ways. First, the effects of actual and perceived similarity on parasocial interactions are compared. Second, similarity is understood in a broad way. With age, gender, job, relationship, and living situation are assessed for sociodemographic similarities. Psychological similarities are considered with the Big Five personality traits, loneliness, and self-esteem.

**Methods:**

The study employs a multimethod design with a field study using tracking data, experience sampling surveys, and content analysis. With the content analysis, characters’ characteristics can be indicated independent from the viewers to assess actual similarity in a more objective way.

**Results:**

In these everyday viewing settings, parasocial interactions increased with similarities in extraversion and perceived Big Five traits and decreased with similarities in age and consciousness. The other assessed similarity types did not influence parasocial interactions.

**Discussion:**

Taken together, the study underlines the importance of differentiating between actual and perceived similarity when analyzing viewer PSI with media characters, and to specify the particular type of similarity.

## 1 Introduction

Similarities between media characters and viewers often explain differences in the strengths of parasocial processes, for example, parasocial interactions (PSI). Parasocial interactions describe an audience member’s illusion of being in a real social interaction with a media character (Hartmann, 2023). Previous research has shown that various similarities influence parasocial interactions. For example, that viewers experience stronger PSI with characters similar in age or gender (Bui, 2017), or similarity in attitudes positively influences their PSI with influencers (Sokolova and Kefi, 2020). Given its importance, it is crucial to understand how similarity is defined in parasocial research: some studies focus only on single characteristics (Sokolova and Kefi, 2020; Greenwood et al., 2021); others on a general similarity (Su et al., 2023); and others on the similarity media users perceive toward the character (Ye et al., 2021; Stein et al., 2022). However, when focusing on single characteristics (e.g., only attitude similarity), it remains unclear if viewers focus on exactly this chosen characteristic for their feeling of similarity. For example, the role of similarity is analyzed by comparing viewers’ age with the character’s age, and the influence of this (dis)similarity on PSI is assessed, without considering other possible similar characteristics between viewer and character (Bui, 2017). When similarity is defined as “general similarity,” a variety of different approaches are used so that behind the same term, different concepts can be hidden. For example, general similarity can mean similar ideas, values, attitudes, preferences or interests (Su et al., 2023). For perceived similarity, only the viewers’ perception is considered, for example, by asking viewers how similar they perceive themselves to an influencer (Stein et al., 2022) or a spokesperson (Ye et al., 2021). Whether viewers and media characters really are similar is not taken into account. This variety in approaches to define and measure similarity makes generalizing the results difficult. Taken together, these different methodological approaches show that comparability of results for the role of similarity in parasocial research becomes more difficult. It underlines the importance of considering these definitions when interpreting the results, as behind the term “similarity,” different ideas can be covered.

Studies on social interactions show that differentiation between *actual* and *perceived* similarity is essential because they can have different effects (Montoya et al., 2008). Actual similarity is defined as the extent to which two individuals share the same characteristics. Perceived similarity describes the extent an individual perceives another person as similar (Wortman et al., 2014). Studies comparing actual and perceived similarity showed a stronger effect of perceived similarity on attraction and on liking than actual similarity (Montoya et al., 2008; Hampton et al., 2019). However, in short interactions between individuals, actual similarity was shown to be essential for the individual’s processing, such as liking or attraction (Montoya et al., 2008). These results underline the importance of differentiating actual and perceived similarity. As a lot of parasocial research derives theoretical assumptions from research about social interactions and relationships, deriving this differentiation to mediated research offers a more nuanced perspective on similarity.

In mediated settings, this distinction between actual and perceived similarity is seldomly made (for exceptions, see Cohen and Hershman-Shitrit, 2017; Webster and Campbell, 2022), and thus, forms a research gap in similarity research in the context of viewers’ PSI with media characters. Based on the research on social interactions, the effects from actual and perceived similarity should be analyzed and compared for viewer PSI with characters. Thus, this research gap is tackled. In a pre-study, the role of actual similarity between viewers and media characters for viewer PSI is tested. As research in social interactions shows that actual similarity was important for individuals’ processing of the encounter (Montoya et al., 2008), it seems worthwhile focusing on actual similarity in the mediated context. However, assessing actual similarity in these mediated settings is challenging. Viewer-perceived similarity can easily be measured by asking participants about how similar they perceive themselves to the media character. Actual similarity needs to be independent from viewers self-assessment, and requires a more objective analysis of media characters sociodemographic or psychological traits to compare them with the self-assessment of viewers.

In this study, actual and perceived similarity are assessed, and the effects of actual and perceived similarity on PSI are compared to integrate this approach of both types of similarities stemming from psychological research also in the mediated setting. Thereby, actual similarity is assessed by independent coders in a content analysis to have a more objective similarity evaluation than viewers’ self-assessment of similarity. The article’s overarching research question is:

*RQ*: How do actual and viewer-perceived similarities between media characters and audience members relate to parasocial interactions?

## 2 Viewers’ parasocial interactions with media characters

Media research has frequently examined how media users connect with, react to, and interact with characters they know through media exposure. Parasocial research broadly focuses on different aspects of viewers’ broad processing of a mediated encounter with a media character. Thereby, PSI describes one specific aspect, differentiating it from other concepts like parasocial relationships or viewers’ identification (Giles, 2023). PSI are restricted to media exposure and defined as an intuitive feeling of mutual awareness, attention, and adjustment with a mediated character (Hartmann, 2023). Viewer PSI is important, as parasocial interactions are crucial for enjoyment and entertainment (Klimmt et al., 2006), or viewers’ information processing and persuasive effects (Rosaen et al., 2019). When viewers interact parasocially, they experience the illusion of being in a real social interaction (Dibble et al., 2023). Due to this illusion, a lot of researchers argue with the theoretical background from social interactions and borrow concepts from social interactions research (Klimmt et al., 2006). For example, traits important in social interactions, such as loneliness, were also analyzed in PSI (Rubin et al., 1985; Lim and Kim, 2011). The influence of similarity on social interactions is adapted to the mediated settings with PSI as interactions between viewers and media characters. Following that, several studies focus on the role of similarity in PSI (Liebers and Schramm, 2019; Schramm et al., 2022). For example, a study showed that perceived similarity with a spokesperson on social media increased customer PSI, which in turn, increased their brand identification (Ye et al., 2021). In another study, similarity was shown to increase viewer PSI with a spokesperson in a public service announcement about obesity. PSI, in turn, increased their intention to adapt their diet and exercise (Phua, 2016). Thus, empirical findings show that similarity can increase PSI, which then possibly impacts other forms of viewers’ message processing like persuasion or entertainment.

PSI is a crucial concept in viewers’ processing of mediated encounters with characters. Understanding viewers’ PSI is important, as PSI has been shown to be essential to viewer entertainment experiences, to persuasion, or to viewers’ message processing (Klimmt et al., 2006; Dibble et al., 2023). Thus, exploring possible predictors of viewer PSI is important to better understand their processing of mediated encounters with characters that are prevalent in all kinds of media productions.

## 3 Actual and viewer-perceived similarity in parasocial interactions

Similarity is an essential predictor of the quality and quantity of social interactions, and the similarity effect is considered the most robust effect in the behavioral sciences (Layton and Insko, 1974). The similarity-attraction hypothesis describes the role of similarity in social interactions and its effects on attraction (Byrne, 1997). It was assumed that individuals sharing similar physical characteristics are generally attracted to each other and that this similarity is thus important for social interactions and relationships (Byrne, 1997). Researchers in psychology distinguish between *actual* and *perceived* similarities (Montoya et al., 2008). Actual similarity has been shown to be important for social interactions and relationships, such as accurately predicting friendship initiation and interactions (AhYun, 2002). A meta-analysis showed that actual similarity was especially important for attraction in short-term interactions, while perceived similarity was important in short-interaction and in existing relationships (Montoya et al., 2008). This underlines that differentiation is necessary, as both concepts are important in other aspects of social interactions and relationships. As in social interactions, both types of similarities are essential, it raises the question about their role in PSI.

The differentiation between actual and perceived similarity is seldom made in media research (for exceptions, see Cohen and Hershman-Shitrit, 2017; Webster and Campbell, 2022), although the assumption that similarity is essential was adapted from social interactions (Klimmt et al., 2006; Greenwood and Aldoukhov, 2023). As research in social interactions shows that actual and perceived similarity can influence different aspects of interactions, it is important to integrate this differentiation also in the mediated context, as they can possibly have different effects on PSI. Most studies analyzing similarity as a predictor of parasocial processes focus on viewer-perceived similarities (Ye et al., 2021; Jhawar et al., 2023). With this approach, it remains open if viewers only perceive them to be similar while being dissimilar. Some studies manipulate similarity based on single characteristics (Hoeken et al., 2016; Igartua and Fiuza, 2018), which approaches actual similarity. With only one similar characteristic, many other characteristics can differ.

To overcome these limitations, two adaptions are made in this study. First, the approach of actual similarity from psychological research is applied to the mediated setting as “objective” similarity. The effects of perceived and actual similarity on PSI are compared. In the study, viewers are asked about how similar they perceive themselves to media characters, and the similarity is additionally assessed in a more objective way to analyze actual similarity. Thus, the effects of both similarity types on PSI can be compared. Second, the similarity is not only limited to a single type of similarity (e.g., gender similarity), but is expanded to several similarity types covering sociodemographic and psychological similarities.

## 4 The role of demographic similarities in parasocial interactions

Similarity is an important predictor of viewer PSI with media characters. Often, it is analyzed viewer-perceived similarities between themselves and the character, which has been shown to increase viewer PSI in several studies, for example, with virtual influencers (Sokolova and Kefi, 2020; Su et al., 2023), with testimonials (Phua, 2016), or with characters in television (Greenwood et al., 2021). Thereby, several studies focus on demographic similarities, for example, the same gender or the same job. As demographic similarity can consist of different attributes (e.g., age, ethnicity) other studies focus on single characteristics describing a specific type of demographic similarity instead of taking all these characteristics together. For example, for shared gender (Hoffner, 1996; Bui, 2017), similarity in age (Bui, 2017), or similarity regarding ethnicity (Pan and Zeng, 2018; Fu et al., 2019), a positive influence on PSI was found. These studies show that different demographic similarities–a general demographic similarity, a perceived similarity, or specific types of demographic similarities– positively influence PSI.

Based on these studies, we expand the analysis of specific types of demographic similarities as predictors for viewer PSI. The influence of similarity in gender and age is retested, and the demographic similarity is further expanded to job, living situation, and relationship status. These additional demographic characteristics were chosen for two reasons. First, these five characteristics are generally essential characteristics for similarity (McPherson et al., 2001), and they are often used in research on parasociality (Liebers and Schramm, 2019). To our knowledge, only the characteristics themselves and their role in PSI (e.g., do singles or married people differ in their PSI?) were analyzed, and not the similarity regarding the relationship status between viewer and media character (e.g., do singles experience stronger PSI with characters also being single than with married characters).

Second, for other forms of viewers’ engagement with characters, these types of similarities were shown to be important. For example, other studies analyzed the influence of job similarity (Hoeken et al., 2016) or of living situation similarity (de Graaf, 2014) on identification. Identification–viewer adaption to a character’s perspective (Cohen, 2001)–differs from PSI, as in identification, viewers merge with the character, and while parasocially interacting, they remain aware of themselves (Dibble et al., 2023). Both concepts describe some sort of viewer engagement with characters (Brown, 2015), so it is worthwhile to retest them for PSI.

By analyzing different specific types of sociodemographic similarity with age, gender, job, living situation and relationship status, this study expands existing research that often focuses on a general sociodemographic similarity or on one single sociodemographic characteristic. Of course, the selection of sociodemographic characteristics with gender, age, job, living situation, and relationship status is non-exhaustive but covers relevant sociodemographic characteristics.

*H1*: Viewers with actual and viewer-perceived demographic backgrounds similar to the media character experience stronger *parasocial interaction* than viewers with different demographic backgrounds.

## 5 The role of psychological similarity in parasocial interactions

Besides sociodemographic factors, psychological factors are important for people’s feeling of similarity in social interactions. For example, personality similarity in the Big Five was shown to result in better initial interaction between two individuals who do not know each other than personality dissimilarity (Cuperman and Ickes, 2009). In social interactions, similarity in personality traits increases the attraction of the interaction partner (Klohnen and Luo, 2003). In a more long-term perspective, similarity in personality traits influenced the partners’ perception of their relationship quality (Gonzaga et al., 2007; Decuyper et al., 2012). These findings show that besides sociodemographics, similarities in traits like the Big Five or other psychological similarities are important in social interactions.

Even though a lot of research on PSI explains the relevance of similarity based on research in social psychology (Klimmt et al., 2006), the role of psychological similarity in mediated encounters is seldom analyzed. One of these studies differentiates between external and internal similarities, with internal similarities as a type of psychological similarity. Both types of similarities had positive effects on PSI, but the effect of internal similarity was stronger than the effect of external similarity (Fu et al., 2019). An example of similarity in a specific psychological trait analyzed with viewers and characters is a study focusing on the Big Five. It showed different effects for the similarity in the basic personality traits (John et al., 2008) and their influence on PSI (Cohen and Hershman-Shitrit, 2017).

The research on psychological similarities in social interactions and the results of the studies about psychological similarities with media characters underline the importance of psychological similarities in social and parasocial interactions. With that comes the need to consider psychological similarities for viewer PSI. In this study, three specific types of psychological similarities and their influence on viewer PSI are analyzed. The selection of the specific psychological traits is explained in the following. Of course, the selection is non-exhaustive but serves as a first step to further explore the relationship between psychological similarities and PSI.

As the first psychological trait to assess the similarity between viewers and media characters, and the influence of this similarity on PSI, the *Big Five* personality traits were chosen. The Big Five consists of openness, conscientiousness, extraversion, agreeableness, and neuroticism (John et al., 2008). They are basic traits used in a lot of research in psychology and in the mediated setting (John et al., 2008). The similarity in the Big Five and its effect on social interactions (Humberg et al., 2023), and in PSI (Cohen and Hershman-Shitrit, 2017) was analyzed in some studies. In the mediated setting, characters from three TV shows were used. The results showed that perceived similarity in the Big Five influenced viewer PSI in the domains of extraversion, conscientiousness, and openness (Cohen and Hershman-Shitrit, 2017). In this study, this relationship is retested with a bigger variety of media characters and with surveys directly after viewers’ media use.

The second chosen personality trait was the viewers’ and the media characters’ *loneliness*. Viewer’s loneliness is often analyzed in the context of PSI (Wang et al., 2008; Lim and Kim, 2011). Researchers often assumed that lonely people compensate for their lack of interpersonal contact with parasocial encounters, but negative or mixed results were found (Rubin et al., 1985; Vorderer and Knobloch, 1996). In this study, the analysis is expanded to the similarity in loneliness between viewer and character, and the role of this similarity in PSI–based on similar research for the role of loneliness similarity in social interactions (Humberg et al., 2023). For example, we want to explore if lonely people experience stronger PSI with a character portrayed as lonely than with a character portrayed as always being around other people.

Third, similarity in *self-esteem* was chosen as a possible predictor for PSI. Self-esteem is another characteristic examined as a predictor of parasocial processes, as it influences how people interact with others (Rosenberg, 1965). In social interactions, an individual’s self-esteem influences their behavior (Burgoon, 1976; McCroskey et al., 1977). In the mediated setting, Turner (1993) showed that viewer self-esteem influences PSI. To our knowledge, there is no research analyzing viewers’ and characters’ similarity in self-esteem on PSI. We want to explore, for example, if viewers with low self-esteem experience stronger PSI with characters also low in self-esteem than with characters with strong self-esteem.

The Big Five (Jackson et al., 2010; Tidwell et al., 2013), loneliness (Stevens and Westerhof, 2006; Mund and Johnson, 2021), and self-esteem (Erol and Orth, 2014, 2016) similarities have been examined in the context of social interactions but not in mediated encounters (Cohen and Hershman-Shitrit, 2017; Webster and Campbell, 2022). They were chosen as these psychological traits are often analyzed in parasocial research. But the similarity between viewer and characters in these traits is seldomly done in the mediated setting, offering a research gap. However, this selection of psychological similarities is not exhaustive, and other characteristics could be important as well. As we cannot derive directed hypotheses for the mediated setting, we formulated the research question:

*RQ2*: How do actual and viewer-perceived similarities between media characters and viewers concerning (a) the Big Five, (b) loneliness, and (c) the level of self-esteem relate to the strength of parasocial interaction?

## 6 Materials and methods

### 6.1 Research design and procedure

A multimethod design assessed the association between similarity for viewers, characters, and PSI. To be as close as possible to participants’ everyday media use, and avoid creating a superficial media use situation, a field study was conducted. This increases the study’s external validity. In this field study, participants’ Netflix use was tracked using a Google Chrome browser extension (Cordeiro et al., 2021). Netflix was chosen as a streaming platform, as a lot of viewers consume media content online and on-demand (Jenner, 2014). The browser extension allows combining usage tracking with survey data. When participants exit Netflix, in a pop-up window, a survey opens, and their thoughts about the previous media use can be assessed directly after watching, avoiding possible memory biases.

Possible participants received information about the study and the use of their data. After signing informed consent, participants installed the browser extension. Then, they completed a general survey that assessed demographics, Big Five, loneliness, and self-esteem. Participants only had to complete this survey once at the beginning of data collection. During the following 2 weeks—always after exiting Netflix—the participants completed a short survey. In this postsession survey, they were asked to select a character from the previously watched content. To generate variance in PSI, they were asked to indicate the participant they thought about the most. This includes liked and disliked media characters and does not limit the selection of media characters to specific types, e.g., protagonists. In each viewing session during these 2 weeks, participants could choose another media character. After indicating the name, the participants’ PSI with and their perceived similarity to this character were assessed. The study was conducted as part of a larger study.^1^

To assess the personality traits of the media characters independent of the study’s participants, a content analysis was conducted. Using the tracking data, information about the watched content was extracted (e.g., series and episode title) and supplemented with the name of the character in the postsession survey. Two coders analyzed the characters the participants chose following a codebook. They indicated the media characters’ age, gender, job, relationship status, living situation, Big Five personality traits, loneliness, and self-esteem. Finally, the data from the usage tracking (what content did they watch?), the postsession surveys (name of the media character, viewer PSI), and the content analysis (media character’s personality traits) were combined into one single data set. The institutional review board of the University of Fribourg approved the study design.

### 6.2 Pre-study

First, a pre-study was conducted to test the multimethod design to assess the association between similarity for viewers, characters, and PSI in an everyday viewing setting (for more details of the methods, see the following chapters of the main study). To do so, the use of Netflix by 80 participants between 18 and 54 years old (*M* = 24.11, *SD* = 8.86) with 69% females was tracked for 2 weeks. After each use, they answered a short survey to assess their PSI with a media character. Participants’ personality traits were assessed in a survey at the beginning of data collection. Coders assessed the media character’s personality traits in a content analysis. With these data, an objectively measured similarity could be assessed. All details of the pre-study are on OSF^2^.

For the pre-study, 425 sessions lasting at least 10 min were tracked. A total of 186 surveys were completed, of which, 156 were completed within the one-hour post-viewing time limit. Sixteen viewing sessions had to be excluded because participants indicated that no characters were relevant. Character choice was required to answer questions about PSI, and thus, sessions without a character had to be excluded. Finally, the data set consisted of 61 participants, with 140 viewing sessions with a selected character.

All items were measured with five-point Likert scales (1 “do not agree at all,” 5 “fully agree”). Participants’ personality traits were assessed in the survey: loneliness (*M* = 1.44, *SD* = 0.45, *α* = 0.82; Russell et al., 1980), self-esteem (*M* = 3.91, *SD* = 0.56, *α* = 0.81; Rosenberg, 1965), Big Five (Rammstedt and John, 2005): extraversion (*M* = 3.65, *SD* = 1.17), agreeableness (*M* = 4.28, *SD* = 0.77), conscientiousness (*M* = 4.06, *SD* = 0.97), neuroticism (*M* = 2.59, *SD* = 1.29), and openness (*M* = 3.90, *SD* = 1.27). In the post-session survey, viewer PSI (*M* = 1.21, *SD* = 0.43, *α* = 0.75) was assessed with the Experience of Parasocial Interaction scale (EPSI; Hartmann and Goldhoorn, 2011).

Characters’ sociodemographics and personality traits were assessed by two coders following a codebook (see 7.1.5 content analysis).Viewers’ loneliness (*M* = 2.99, *SD* = 1.01, *α* = 0.96), self-esteem (*M* = 3.81, *SD* = 0.65, *α* = 0.95) Big Five: extraversion (*M* = 3.50, *SD* = 1.18), agreeableness (*M* = 2.93, *SD* = 1.08), conscientiousness (*M* = 3.77, *SD* = 0.93), neuroticism (*M* = 2.70, *SD* = 1.05), and openness (*M* = 3.49, *SD* = 0.90) were assessed each in one category based on the items used in the surveys. The reliability test of 10 randomly selected cases showed satisfactory results: the averages over all items for each category are for sociodemographic *α* = 1, Big Five *α* = 0.780, loneliness *α* = 0.857, and self-esteem *α* = 0.797 (for all single items on OSF).

Based on viewers’ self-assessment in the surveys, and coders’ assessment of the characters, similarity scores were calculated. The results (see OSF for more details) showed only one significant predictor for PSI. Viewers rated as similar in extraversion experienced stronger PSI than viewers dissimilar in extraversion to the character.

The pre-study disclosed two points that needed to be considered in the main study. First, only actual similarity was analyzed. It could be assumed that actual similarity cannot create the sort of intimacy that is crucial for PSI (Klimmt et al., 2006). The interpretation was limited without information about participant-perceived similarities; thus, it is unclear whether viewers did not feel similar or felt similar despite being objectively dissimilar.

Second, the EPSI scale used was not ideal in the study setting. The scale was developed for a TV host addressing viewers through verbal or physical cues (Hartmann and Goldhoorn, 2011). In this study, participants watched content on Netflix wherever they wanted and interacted with various characters. In these self-directed viewing sessions, the PSI intensities were very low (EPSI, *M* = 1.21, *SD* = 0.43).

The use of the multimethod design was proven to be useful in the pre-study. However, the limitations of the pre-study led to the additional assessment of viewer-perceived similarity in the main study, and the use of another scale to assess viewer PSI to increase the validity of the main study.

### 6.3 Participants

For the main study, 100 individuals with access to Netflix were recruited at the University of Fribourg. The participants were between 18 and 38 years old (*M* = 23.14, *SD* = 4.13), with 81% identifying themselves as female, 17% as male, and 1% as nonbinary. Students (45%) and part-time employees (40%) accounted for the largest portion of the sample. Most participants (97%) were single.

### 6.4 Usage tracking and survey data

In total, 693 viewing sessions of 94 participants were tracked. Sessions were excluded when the survey was completed too late (>1 h after watching, *n* = 40), sessions lasted less than 10 min (*n* = 28), or viewers did not choose a character due to a lack of relevant characters (*n* = 203). During content analysis, additional sessions were excluded (*n* = 40) for several reasons, such as when the named character did not match the watched content, the character was nonspecific (e.g., “the girl”), or several characters were named (e.g., Ginny and Georgia). These exclusions resulted in a final sample of 317 sessions of 91 participants.

### 6.5 Measures

Unless otherwise mentioned, all items were measured using a five-point Likert-type scale ranging from 1 (“do not agree at all”) to 5 (“fully agree”). The full scales are on OSF.

#### 6.5.1 Loneliness

Participant loneliness levels (*M* = 1.64, *SD* = 0.66, *α* = 0.88) were assessed with six items of the revised UCLA Loneliness Scale (e.g., “I feel left out”; Russell et al., 1980).

#### 6.5.2 Self-esteem

Participant self-esteem levels (*M* = 3.60, *SD* = 0.79, *α* = 0.90) were measured using 10 items (e.g., “I wish I could have more respect for myself”; Rosenberg, 1965).

#### 6.5.3 Big Five

Each participant’s personality profile was assessed using one item of the German short form of the Big Five Inventory scale for each of the five dimensions (Rammstedt and John, 2005): extraversion, (*M* = 3.67, *SD* = 1.06); agreeableness, (*M* = 3.26, *SD* = 1.06); conscientiousness, (*M* = 4.08, *SD* = 0.73); neuroticism, (*M* = 3.36, *SD* = 1.13); and openness, (*M* = 3.67, *SD* = 1.30). The use of single-item measures is common in experience sampling studies and was proven to result in valid measurements in other studies (Allen et al., 2022; Matthews et al., 2022).

#### 6.5.4 PSIs

Viewer PSI was measured using six items from the PSI-Process scale (Schramm and Hartmann, 2008), which measures viewer PSI (*M* = 2.58, *SD* = 0.87, *α* = 0.72), defined as the “degree to which the individual interacts psychologically with a media character” (p. 388). The scale is applicable to a variety of media characters, and applicable also in these self-directed and everyday viewing sessions.

#### 6.5.5 Perceived similarity

To compare perceived and actual similarity, participants were asked to indicate in the postsession survey how similar they perceived themselves to be to the character. Viewers self-assessing their similarity to the character is based on other research in parasocial research (Fu et al., 2019; Stein et al., 2022). As in these studies, only a general perceived similarity was assessed, we formulated own items for each type of perceived similarity: sociodemographic similarity (*M* = 1.93, *SD* = 1.19), Big Five (*M* = 2.63, *SD* = 1.24), loneliness (*M* = 2.40, *SD* = 1.27), and self-esteem (*M* = 2.81, *SD* = 1.22). For example, to assess viewer-perceived similarity in self-esteem, participants indicated how much the statement “Character and I are very similar in terms of our level of self-esteem” applied to them. The participants were instructed about how they can get more information about terms they might not understand (e.g., conscientiousness), and could move the mouse in the online survey on the word, and in a pop-up box a short explanation appeared to make it easier for them to answer the questions.

### 6.6 Content analysis

The content analysis sample was determined by the characters chosen in the 140 postsession surveys. Four further sessions were excluded because the chosen characters did not match the watched content (*n* = 136, e.g., a participant watched *The Big Bang* Theory and named James Bond in the postsession survey). Two coders assessed the chosen characters following a codebook^3^ and according to the *state of play* indicated by the watched episode. Characters evolve during a series, potentially over long periods of the characters’ lives. For example, in the first episode of *Gilmore Girls*, Rory is coded as a shy 16-year-old student. In the last episode of the final season, Rory is coded as an outgoing young woman starting her career.

The items from the surveys were used as categories in the content analysis. The coders rated the items’ appropriateness for the character from 1 (“not applicable at all”) to 5 (“totally applicable”). For example, they indicated whether the sentence “There are people who really understand CHARACTER” applied to assess the character’s loneliness.

For 10 randomly selected cases, intercoder reliability was calculated with Krippendorff’s alpha > = 0.667 (Krippendorff, 2004). The reliability test showed satisfactory results: the averages over all items for each category are for sociodemographic *α* = 0.99, Big Five *α* = 0.803, loneliness *α* = 0.816, and self-esteem *α* = 0.741 (more detailed on OSF).

#### 6.6.1 Sociodemographics

Each character’s gender (male/female/non-binary), age (in 10-year increments), job (employed, unemployed, student/training), living situation (alone, with a partner, in a shared apartment, with family), and relationship status (single, married, divorced, widowed) was assessed. An additional option (“does not apply”) was eligible for all variables. For example, some fictional characters do not have ordinary jobs, and characters in prison do not match the listed living situations.

#### 6.6.2 Loneliness

Character loneliness (*M* = 2.66, *SD* = 0.92, *α* = 0.94) was assessed based on six items from the UCLA Loneliness Scale (Russell et al., 1980).

#### 6.6.3 Self-esteem

Character self-esteem (*M* = 3.69, *SD* = 98, *α* = 0.95) was assessed using the Rosenberg Self-Esteem Scale (Rosenberg, 1965).

#### 6.6.4 Big Five

Each character’s personality profile was assessed using one item of the German short form of the Big Five Inventory scale for each of the five dimensions (Rammstedt and John, 2005): extraversion (*M* = 3.61, *SD* = 1.18), agreeableness (*M* = 3.21, *SD* = 1.25), conscientiousness (*M* = 3.87, *SD* = 1.07), neuroticism (*M* = 2.61, *SD* = 1.07), and openness (*M* = 3.71, *SD* = 1.09).

### 6.7 Similarity between audiences and media characters

For the sociodemographic variables and the Big Five, similarity scores were calculated by adding one point if character and viewer matched (e.g., same gender) and zero points if they did not match.

For loneliness and self-esteem, similarity was calculated by subtracting the character value from the participant value for each item. The absolute values of those similarity scores were then used to build a mean index for all items (6 items for loneliness and 10 items for self-esteem). These overall similarity indices were recoded from 0 (“very dissimilar”) to 4 (“very similar”).

Adding up the different similarity scores was modeled on the procedure used in studies of actual similarity in personal interactions and relationships (e.g., Tidwell et al., 2013; van Zalk and Denissen, 2015). Both similarity scores relied on viewers rating their demographics and characteristics in the same manner as the coders who rated character demographics and characteristics. In both cases, measurement invariance exists, so we refrained from considering a score of 4 as “completely similar.” As participants’ self-assessment of their sociodemographics and personality traits were used, we refrained from calling it “actual” similarity, as the measurements are not free from subjective influences.

## 7 Results

Participants engaged in one to 23 viewing sessions (*M* = 3.98, *SD* = 3.95). Multilevel regressions were calculated using the R package *lme4*. The null models showed that PSI (ICC = 48%) depended to a significant extent on the viewing session and, thus, possibly on the characters.

To test H1, a multilevel model was calculated using demographic similarity scores (Table 1). Viewers with a similar age experienced weaker PSI (*β* = −0.28, *SE* = 0.11, *p* = 0.010), but viewers with a similar relationship status experienced stronger PSI (*β* = 0.30, *SE* = 0.10, *p* = 0.003). Similarities in gender, job, or living situation did not influence PSI. Viewers’ perceived demographic similarity also did not influence their PSI. Thus, H1 was confirmed for relationship similarity. For the other actual similarities and perceived similarity, H1 was rejected. Compared to the null model, the demographic similarities only explained R^2^_1_ = 0.002 variance in PSI (Snijders and Bosker, 2012).

**Table 1:** The influence of demographic similarities on parasocial interactions.

	Parasocial interactions		
Predictors	Est.	SE	*p*
Intercept	3.47	0.12	<0.001
age similarity	−0.28	0.11	0.010
gender similarity	0.02	0.08	0.817
relationship similarity	0.30	0.10	0.003
job similarity	−0.02	0.11	0.838
living situation similarity	0.16	0.13	0.219
perceived demographic similarity	0.06	0.04	0.151
Random effects			
σ^2^	0.27		
τ_00_	0.24 _User_		

Actual similarities: 0 = dissimilar, 1 = similar. Perceived similarity: 0 = dissimilar to 5 = similar. *N* = 78 participants with 233 viewing sessions. R^2^_1_ (R-square of level 1 predictors following Snijders and Bosker (2012)) = 0.002.

RQ2 was tested with a multilevel model using all psychological similarities (Table 2). Regarding the Big Five similarities, different patterns emerged for the five dimensions. Viewers with actual extraversion similarity (*β* = 0.23, *SE* = 0.09, *p* = 0.015) experienced stronger PSI, whereas viewers with actual consciousness similarity (*β* = −0.20, *SE* = 0.08, *p* = 0.019) indicated lower PSI. The perceived similarity regarding the Big Five positively influenced their PSI (*β* = 0.08, *SE* = 0.04, *p* = 0.039). To answer RQ2, the degree to which viewers perceived themselves to be similar to the character significantly increased their PSI. Regarding actual similarity, differences emerged for the five domains, with extraversion similarity increasing PSI and consciousness similarity decreasing PSI.

**Table 2:** The influence of similarity in psychological similarities on parasocial interactions.

	Parasocial interactions		
Predictors	Est.	SE	*p*
Intercept	3.76	0.23	<0.001
extraversion similarity	0.23	0.09	0.015
agreeableness similarity	0.08	0.09	0.397
consciousness similarity	−0.20	0.08	0.019
neuroticism similarity	−0.06	0.08	0.471
openness similarity	0.00	0.08	0.953
perceived personality similarity	0.08	0.04	0.039
loneliness similarity	−0.09	0.05	0.092
perceived similarity in loneliness	−0.01	0.04	0.736
self-esteem similarity	−0.01	0.07	0.877
perceived similarity in self-esteem	0.02	0.04	0.532
Random effects			
σ^2^	0.25		
τ_00_	0.26 _User_		

Actual similarities: 0 = dissimilar, 1 = similar. Perceived similarity: 0 = dissimilar to 5 = similar. *N* = 84 participants with 296 viewing sessions. R^2^_1_ (R-square of level 1 predictors following Snijders and Bosker (2012)) = 0.04.

Regarding RQ2b for loneliness, actual similarity (*β* = −0.09, *SE* = 0.05, *p* = 0.092), and the perceived loneliness similarity both did not influence the degree of viewer PSI (*β* = −0.01, *SE* = 0.04, *p* = 0.736). The same was found for RQ2c for self-esteem. Actual assessed similarity (*β* = −0.01, *SE* = 0.07, *p* = 0.877) and perceived self-esteem similarity both did not influence PSI (*β* = 0.02, *SE* = 0.04, *p* = 0.532). Compared to the null model, the psychological similarities explained R^2^_1_ = 0.04 variance in PSI (Snijders and Bosker, 2012).

## 8 Discussion

Similarity between viewers and media characters has been examined frequently in research on PSI (Phua, 2016; Bui, 2017). To expand this literature, this study applies an approach that differentiates actual and perceived similarity in a mediated setting (Montoya et al., 2008). Existing research often focuses on single characteristics (Sokolova and Kefi, 2020; Greenwood et al., 2021) or a general similarity (Su et al., 2023) between viewers and characters. This study broadly covered similarities by including different sociodemographic and psychological similarities. Furthermore, viewers’ media use was analyzed in everyday viewing settings, resulting in high external validity. By comparing the effects of actual similarity and perceived similarity broadens the existing literature about similarity in mediated interactions. Taken together, the results of our study underline the need to apply a nuanced approach to analyses of similarity by differentiating between similarity types (e.g., sociodemographic, psychological) and between the similarities viewers perceive and actual similarities to better cover the concept of similarity.

For viewers’ similarity with the media characters, their perceived demographic similarity did not influence PSI. However, actual relationship similarity increased PSI, whereas actual age similarity decreased it. This contradicts another study that found a positive effect between age similarity and viewer PSI with their favorite celebrities (Bui, 2017). In our study, viewers indicated their PSI with mostly fictional characters on Netflix. It is possible that age and sociodemographic similarities are less important with fictional characters than with real people (Hartmann and Hofer, 2021). Overall, these findings challenge existing literature, as mostly, similar demographic background did not increase viewer PSI in these everyday viewing settings. Possibly, in everyday self-directed viewing sessions, similarity is less important for viewer PSI than, for example, in a single exposure situation with a public service announcement.

Several studies on social interactions (Klohnen and Luo, 2003; Cuperman and Ickes, 2009) and one study on mediated encounters (Fu et al., 2019) showed the importance of psychological similarity. This assumption was retested in the mediated setting. For the Big Five, actual similar viewers in extraversion experienced stronger PSI. In the context of mostly fictional Netflix content, character extraversion might be prevalent. As a result, this personality trait is obvious to the viewers, and similarity in extraversion, thus, relevant for viewer PSI. Viewers similar in consciousness indicated lower PSI, which might mean that viewers with high consciousness like to engage with dissimilar characters to escape from their familiar everyday lives.

Regarding loneliness and self-esteem similarity, no effect of actual and perceived similarity was found. In social interactions, the positive effect of psychological similarity on interactions was mainly proven in hypothetical settings (Montoya et al., 2008). In real-life social interactions, several studies do not find evidence for an influence of psychological similarity on social interactions (Luo and Zhang, 2009; Asendorpf et al., 2011; Tidwell et al., 2013). Viewers’ interactions with characters come closer to hypothetical interactions, as they are one-sided and take place in a limited setting (Hartmann and Goldhoorn, 2011), aligning with these results of social interactions.

By comparing the actual and perceived similarities between viewers and characters, this study expands the existing literature. Only perceived Big Five similarities positively influenced viewer PSI. This result aligns with existing findings in the mediated settings (Cohen and Hershman-Shitrit, 2017). The additionally analyzed perceived similarities in sociodemographics, loneliness, and self-esteem did not influence PSI. One explanation is that perceived similarity was important in existing relationships of individuals, whereas actual similarity was only important in short-term interactions (Montoya et al., 2008). Following this reasoning, perceived similarity would be essential for parasocial relationships–viewers’ long-term psychological involvement with a character (Dibble et al., 2023)–but not for the short-term oriented PSI (Hartmann, 2023).

It is important to also consider the relationship between actual and perceived similarity. Cohen and Hershman-Shitrit (2017) showed that these two measurements can differ, and so do their effects. Therefore, the measurement of both types of similarity is essential. The items to assess viewer-perceived similarity were based on other studies in parasocial research (e.g., Fu et al., 2019; Stein et al., 2022) but adapted to the specific types of similarity assessed in this study, e.g., demographic or loneliness similarity. These author-developed items are somehow limited, as in one item, several comparisons are covered. For example, viewers indicated in only one item if they perceive themselves to be similar to the character regarding the personality traits that cover openness, extraversion, neuroticism, agreeableness, and conscientiousness. If viewers perceive themselves as similar in extraversion but not in openness, it is hard to answer this item, and the validity of the measurement can be questioned. Thus, the lack of influence of viewer-perceived similarity on parasocial interactions needs to be interpreted with caution. It is possible that the measurement used in the study was not able to capture viewers’ feelings of similarity, and thus, the lack of significant findings represents an inadequate measurement rather than a lack of influence. In future studies, it would be important to improve this similarity measurement. For example, by letting viewer evaluate their perceived similarity in the Big Five for each of the five dimensions separately.

Taken together, this study adds to the existing literature in several ways. First, it provides new empirical findings for the relationship between actual and perceived similarity and their influence on PSI. The assessment of both types of similarities follows the approach of research in social interactions in the mediated setting. Second, the different results for similarity in the Big Five confirm existing results (Cohen and Hershman-Shitrit, 2017) and underline that only actual similarity increases viewer PSI but not perceived similarity. This finding challenges other studies’ findings that only focused on viewer-perceived similarity and concluded that similarity is unimportant for PSI. Our study’s result can provide an explanation for different findings when experimentally creating an actual similarity (e.g., by choosing a protagonist with the same gender) or when asking viewers about their perceived similarity.

The findings suggest some adaptions in future research about similarity in mediated interactions between viewers and media characters. In future studies, it should be either clearly labeled what type of similarity is assessed (actual vs. perceived). To see if both types of similarity have the same effects on interactions as shown in research about social interactions (Montoya et al., 2008), both types of similarity should be assessed, and their effects on PSI compared in different mediated settings, for example, with influencers on social media, celebrities in television, or fictional characters in entertainment productions. Thereby, it could be analyzed if this differentiation is important for all interactions or only for specific settings (e.g., social media, television), or only with specific media characters (e.g., real celebrities, fictional characters). For parasocial research overall, future studies about the actual and perceived similarity between viewers and media characters could advance the understanding of how and why viewers interact parasocially with different types of characters in various mediated settings.

### 8.1 Limitations

This study has some limitations. First, actual similarity measurement was not protected against subjective influences because viewer personality traits were collected by self-assessment. If participants did not want to identify as lonely, they might have adapted their self-assessment. Another concern is that participant loneliness and self-esteem were measured only at the beginning of the 2 weeks of data collection. Future studies might consider assessing viewer states of loneliness and self-esteem at each viewing situation.

Second, this study limited similarity measurements to a non-exhaustive set of characteristics: sociodemographics, the Big Five, loneliness, and self-esteem. Other factors might have been more critical to viewer similarity evaluations than the analyzed characteristics. An individual may share similar physical characteristics with a character but have different values, which might be more relevant to viewer similarity perceptions. The power of media often lies in its ability to create empathy for characters who have experiences that are different from those of viewers (Wong et al., 2017). Individuals are multifaceted, and similarity as a measure might be an oversimplification.

Third, the watched content was not considered. Participants were free to watch whatever, and however much they wanted. This advantage, in terms of external validity, resulted in participants watching various genres. We did not control for genres, usage situations, or attention. Depending on that, different characteristics of the characters might have been prevalent. In a legal drama, moral similarity might be more relevant than in a comedy show. In this study’s entertainment context, actual similarity was not important for viewer PSI; however, it might be important in other media types (e.g., news reports).

Fourth, only the viewing sessions of participants who filled in the postsession survey were included in the analysis. This was necessary to gather data about the chosen characters. This resulted in a relatively small sample size, and with that, a rather low power to detect the proposed effects. This rather low power is important to keep in mind when interpreting the null effects of this study. Post-hoc power analyses following Scherbaum and Ferreter (2009) showed that, in general in a multilevel regression, for 100 participants with, on average, three viewing sessions during 2 weeks, a medium effect (*d* = 0.30) with a medium variance (*ρ* = 0.1) would result in a power of 0.66 for this study. To achieve sufficient power (> 0.80) in future studies with a comparable design, either 140 participants would be needed, or the use of 90 participants would need to be tracked for 4 weeks (more details on this issue can be found on OSF). Additionally, in our study, we might have lost data about intense viewing sessions, as intense viewing might have caused viewers to fail to complete the survey because they were too distracted. A more extended period of tracking data would facilitate an analysis of development over time. For example, a viewer’s Netflix use might be tracked when a new season of a show featuring their favorite character is released to analyze their PSI after each new episode they watch. Accordingly, the role of similarity might be considered in repeated PSI with the same character (Gleich, 1996).

### 8.2 Conclusion

This study contributes to the existing literature about the role of similarity in viewer PSI in several ways. First, in this mediated setting, the study distinguished between actual and subjective similarity, which research on social interactions has deemed important (Montoya et al., 2008). Second, the study analyzed the role of similarity in the context of viewers’ everyday media use through a field study that combined tracking and survey data. Third, similarities were analyzed from a broad perspective, including different demographic and psychological similarities. In everyday viewing situations, viewer PSI increased with extraversion similarity and perceived personality trait similarity but decreased with age and consciousness similarities. The other actual and perceived similarity types did not influence viewer PSI. Future studies should specify which type of similarity is being analyzed and in which way similarity is assessed, respecting the difference between actual and subjective measures.

## Data availability statement

The data supporting this study’s findings are openly available in OSF: https://osf.io/yz3rq/.

## Ethics statement

The study involving humans was approved by Institutional Review Board for research ethics (IRB), Faculty of Management, Economics and Social Sciences, University of Fribourg. The study was conducted in accordance with the local legislation and institutional requirements. The participants provided their written informed consent to participate in this study.

## Author contributions

MM: Conceptualization, Data curation, Formal analysis, Investigation, Methodology, Project administration, Writing – original draft, Writing – review & editing. AF: Conceptualization, Methodology, Supervision, Validation, Writing – review & editing.

## Footnotes

^1^ See https://osf.io/ceb9n/.

^2^https://osf.io/yz3rq/

^3^https://bit.ly/3j5BRaK

## References

[ref1] AhYun K. (2002). “Similarity and attraction” in Interpersonal communication research. eds. Allen M. Preiss R. W. Gayle B. M. Burrell N. A. (Mahwah, NJ: Erlbaum), 145–167.

[ref2] Allen M. S. Iliescu D. Greiff S. (2022). Single item measures in psychological science. Eur. J. Psychol. Assess. 38, 1–5. doi: 10.1027/1015-5759/a000699

[ref3] Asendorpf J. B. Penke L. Back M. D. (2011). From dating to mating and relating: predictors of initial and long–term outcomes of speed–dating in a community sample. Eur. J. Personal. 25, 16–30. doi: 10.1002/per.768

[ref4] Brown W. J. (2015). Examining four processes of audience involvement with media personae: transportation, parasocial interaction, identification, and worship: examining four processes of audience involvement with media personae. Commun. Theory 25, 259–283. doi: 10.1111/comt.12053

[ref5] Bui N. H. (2017). Exploring similarity characteristics, identification, and parasocial interactions in choice of celebrities. Psychol. Pop. Media Cult. 6, 21–31. doi: 10.1037/ppm0000082

[ref6] Burgoon J. K. (1976). The unwillingness-to-communicate scale: development and validation. Commun. Monogr. 43, 60–69. doi: 10.1080/03637757609375916

[ref7] Byrne D. (1997). An overview (and underview) of research and theory within the attraction paradigm. J. Soc. Pers. Relat. 14, 417–431. doi: 10.1177/0265407597143008

[ref8] Cohen J. (2001). Defining identification: a theoretical look at the identification of audiences with media characters. Mass Commun. Soc. 4, 245–264. doi: 10.1207/S15327825MCS0403_01

[ref9] Cohen J. Hershman-Shitrit M. (2017). Mediated relationships with TV characters. Sci. Study Lit. 7, 109–128. doi: 10.1075/ssol.7.1.05coh

[ref10] Cordeiro J. A. Castro D. Nisi V. Nunes N. J. (2021). BWDAT: a research tool for analyzing the consumption of VOD content at home. Addict. Behav. Rep. 13:100336. doi: 10.1016/j.abrep.2020.100336, PMID: 33644293 PMC7889796

[ref11] Cuperman R. Ickes W. (2009). Big five predictors of behavior and perceptions in initial dyadic interactions: personality similarity helps extraverts and introverts, but hurts “Disagreeables.”. J. Pers. Soc. Psychol. 97, 667–684. doi: 10.1037/a0015741 19785485

[ref12] de Graaf A. (2014). The effectiveness of adaptation of the protagonist in narrative impact: similarity influences health beliefs through self-referencing. Hum. Commun. Res. 40, 73–90. doi: 10.1111/hcre.12015

[ref13] Decuyper M. De Bolle M. De Fruyt F. (2012). Personality similarity, perceptual accuracy, and relationship satisfaction in dating and married couples. Pers. Relat. 19, 128–145. doi: 10.1111/j.1475-6811.2010.01344.x

[ref14] Dibble J. L. Tukachinsky Forster R. Guzaitis M. Downey S. E. (2023). “Methods and measures in investigating PSEs” in The Oxford handbook of Parasocial experiences. ed. Tukachinsky Forster R. (Oxford: Oxford University Press)

[ref15] Erol R. Y. Orth U. (2014). Development of self-esteem and relationship satisfaction in couples: two longitudinal studies. Dev. Psychol. 50, 2291–2303. doi: 10.1037/a0037370 24999764

[ref16] Erol R. Y. Orth U. (2016). Self-esteem and the quality of romantic relationships. Eur. Psychol. 21, 274–283. doi: 10.1027/1016-9040/a000259

[ref17] Fu S. Xu Y. Yan Q. (2019). Enhancing the parasocial interaction relationship between consumers through similarity effects in the context of social commerce: evidence from social commerce platforms in China. J. Strateg. Mark. 27, 100–118. doi: 10.1080/0965254X.2017.1384045

[ref18] Giles D. (2023). “Defining Parasocial relationship experiences” in The Oxford handbook of Parasocial experiences. ed. Tukachinsky Forster R. (Oxford: Oxford University Press)

[ref19] Gleich U. (1996). “Sind Fernsehpersonen die Freunde des Zuschauers? Ein Vergleich zwischen parasozialen und realen sozialen Beziehungen” in Fernsehen als Beziehungskiste. ed. Vorderer P. (Opladen: VS Verlag für Sozialwissenschaften), 113–144.

[ref20] Gonzaga G. C. Campos B. Bradbury T. (2007). Similarity, convergence, and relationship satisfaction in dating and married couples. J. Pers. Soc. Psychol. 93, 34–48. doi: 10.1037/0022-3514.93.1.34, PMID: 17605587

[ref21] Greenwood D. Aldoukhov A. (2023). “The social context of PSRs” in The Oxford handbook of Parasocial experiences. ed. Tukachinsky Forster R. (Oxford: Oxford University Press)

[ref22] Greenwood D. Ribieras A. Clifton A. (2021). The dark side of antiheroes: antisocial tendencies and affinity for morally ambiguous characters. Psychol. Pop. Media 10, 165–177. doi: 10.1037/ppm0000334

[ref23] Hampton A. J. Fisher Boyd A. N. Sprecher S. (2019). You’re like me and I like you: mediators of the similarity–liking link assessed before and after a getting-acquainted social interaction. J. Soc. Pers. Relat. 36, 2221–2244. doi: 10.1177/0265407518790411

[ref24] Hartmann T. (2023). “Three conceptual challenges to Parasocial interaction: anticipated responses, implicit address, and the interactivity problem” in The Oxford handbook of Parasocial experiences. ed. Tukachinsky Forster R. (Oxford: Oxford University Press)

[ref25] Hartmann T. Goldhoorn C. (2011). Horton and Wohl revisited: exploring viewers’ experience of parasocial interaction. J. Commun. 61, 1104–1121. doi: 10.1111/j.1460-2466.2011.01595.x

[ref26] Hartmann T. Hofer M. (2021). I know it is not real (and that matters): media awareness vs. presence shape the VR experience. PsyArXiv 2021, 1–32. doi: 10.31234/osf.io/a2ykd

[ref27] Hoeken H. Kolthoff M. Sanders J. (2016). Story perspective and character similarity as drivers of identification and narrative persuasion. Hum. Commun. Res. 42, 292–311. doi: 10.1111/hcre.12076

[ref28] Hoffner C. (1996). Children’s wishful identification and parasocial interaction with favorite television characters. J. Broadcast. Electron. Media 40, 389–402. doi: 10.1080/08838159609364360

[ref29] Humberg S. Gerlach T. M. Franke-Prasse T. Geukes K. Back M. D. (2023). Is (actual or perceptual) personality similarity associated with attraction in initial romantic encounters? A dyadic response surface analysis. Pers. Sci. 4, 1–25. doi: 10.5964/ps.7551

[ref30] Igartua J.-J. Fiuza D. (2018). Persuading with narratives against gender violence. Effect of similarity with the protagonist on identification and risk-perception. Palabra Clave 21, 499–523. doi: 10.5294/pacla.2018.21.2.10

[ref31] Jackson B. Dimmock J. A. Gucciardi D. F. Grove J. R. (2010). Relationship commitment in athletic dyads: actor and partner effects for big five self- and other-ratings. J. Res. Pers. 44, 641–648. doi: 10.1016/j.jrp.2010.08.004

[ref32] Jenner M. (2014). Is this TVIV? On Netflix, TVIII and binge-watching. New Media Soc. 18, 257–273. doi: 10.1177/1461444814541523

[ref33] Jhawar A. Kumar P. Varshney S. (2023). The emergence of virtual influencers: a shift in the influencer marketing paradigm. Young Consum. 24, 468–484. doi: 10.1108/YC-05-2022-1529

[ref34] John O. P. Naumann L. P. Soto C. J. (2008). “Paradigm shift to the integrative big-five trait taxonomy. History, measurement, and conceptual issues” in Handbook of personality: Theory and research. eds. John O. P. Robins R. W. Pervin L. A. (New York: Guildford Press), 114–158.

[ref35] Klimmt C. Hartmann T. Schramm H. (2006). “Parasocial interactions and relationships” in Psychology of entertainment. eds. Bryant J. Vorderer P. (Mahwah, NJ: Lawrence Erlbaum Associates Publishers), 291–313.

[ref36] Klohnen E. C. Luo S. (2003). Interpersonal attraction and personality: what is attractive--self similarity, ideal similarity, complementarity or attachment security? J. Pers. Soc. Psychol. 85, 709–722. doi: 10.1037/0022-3514.85.4.709 14561124

[ref001] Krippendorff K. (2004). Reliability in content analysis. Some common misconceptions and recommendations. Human Comm. Res. 30, 411–433. doi: 10.1111/j.1468-2958.2004.tb00738.x

[ref37] Layton B. D. Insko C. A. (1974). Anticipated interaction and the similarity-attraction effect. Sociometry 37, 149–162. doi: 10.2307/2786372

[ref38] Liebers N. Schramm H. (2019). Parasocial interactions and relationships with media characters - an inventory of 60 years of research. Commun. Res. Trends 38, 4–31.

[ref39] Lim C. M. Kim Y.-K. (2011). Older consumers’ TV home shopping: loneliness, parasocial interaction, and perceived convenience. Psychol. Mark. 28, 763–780. doi: 10.1002/mar.20411

[ref40] Luo S. Zhang G. (2009). What leads to romantic attraction: similarity, reciprocity, security, or beauty? Evidence from a speed-dating study. J. Pers. 77, 933–964. doi: 10.1111/j.1467-6494.2009.00570.x, PMID: 19558447

[ref41] Matthews R. A. Pineault L. Hong Y. (2022). Correction to: normalizing the use of single-item measures: validation of the single-item compendium for organizational psychology. J. Bus. Psychol. 37:873. doi: 10.1007/s10869-022-09816-0

[ref42] McCroskey J. C. Daly J. A. Richmond V. P. Falcione R. L. (1977). Studies of the relationship between communication apprehension and self-esteem. Hum. Commun. Res. 3, 269–277. doi: 10.1111/j.1468-2958.1977.tb00525.x

[ref43] McPherson M. Smith-Lovin L. Cook J. M. (2001). Birds of a feather: homophily in social networks. Annu. Rev. Sociol. 27, 415–444. doi: 10.1146/annurev.soc.27.1.415

[ref44] Montoya R. M. Horton R. S. Kirchner J. (2008). Is actual similarity necessary for attraction? A meta-analysis of actual and perceived similarity. J. Soc. Pers. Relat. 25, 889–922. doi: 10.1177/0265407508096700

[ref45] Mund M. Johnson M. D. (2021). Lonely me, lonely you: loneliness and the longitudinal course of relationship satisfaction. J. Happiness Stud. 22, 575–597. doi: 10.1007/s10902-020-00241-9

[ref46] Pan P.-L. Zeng L. (2018). Parasocial interactions with basketball athletes of color in online mediated sports. Howard J. Commun. 29, 196–215. doi: 10.1080/10646175.2017.1354790

[ref47] Phua J. (2016). The effects of similarity, parasocial identification, and source credibility in obesity public service announcements on diet and exercise self-efficacy. J. Health Psychol. 21, 699–708. doi: 10.1177/1359105314536452 24925545

[ref48] Rammstedt B. John O. P. (2005). Kurzversion des big five inventory (BFI-K). Diagnostica 51, 195–206. doi: 10.1026/0012-1924.51.4.195

[ref49] Rosaen S. F. Dibble J. L. Hartmann T. (2019). Does the experience of parasocial interaction enhance persuasiveness of video public service messages? Commun. Res. Rep. 36, 201–208. doi: 10.1080/08824096.2019.1598854

[ref50] Rosenberg M., (1965). Society and the adolescent self-image. Princeton University Press, Princeton.

[ref51] Rubin A. M. Perse E. M. Powell R. A. (1985). Loneliness, parasocial interaction, and local television news viewing. Hum. Commun. Res. 12, 155–180. doi: 10.1111/j.1468-2958.1985.tb00071.x

[ref52] Russell D. Peplau L. A. Cutrona C. E. (1980). The revised UCLA loneliness scale: concurrent and discriminant validity evidence. J. Pers. Soc. Psychol. 39, 472–480. doi: 10.1037//0022-3514.39.3.472, PMID: 7431205

[ref53] Scherbaum C. A. Ferreter J. M. (2009). Estimating statistical power and required sample sizes for organizational research using multilevel modeling. Organ. Res. Methods 12, 347–367. doi: 10.1177/1094428107308906

[ref54] Schramm H. Hartmann T. (2008). The PSI-process scales. A new measure to assess the intensity and breadth of parasocial processes. Communications 33, 385–401. doi: 10.1515/COMM.2008.025

[ref55] Schramm H. Liebers N. Biniak L. Dettmar F., (2022). Neuere Forschung zu parasozialen Interaktionen und Beziehungen, Nomos Verlagsgesellschaft. Waldseestraße

[ref56] Snijders T. A. B. Bosker R. J., (2012). Multilevel analysis: An introduction to basic and advanced multilevel modeling, second. SAGE Publications Ltd, Cham.

[ref57] Sokolova K. Kefi H. (2020). Instagram and YouTube bloggers promote it, why should I buy? How credibility and parasocial interaction influence purchase intentions. J. Retail. Consum. Serv. 53:101742. doi: 10.1016/j.jretconser.2019.01.011

[ref58] Stein J.-P. Breves P. L. Anders N. (2022). Parasocial interactions with real and virtual influencers: the role of perceived similarity and human-likeness. New Media Soc. 900:14614448221102900. doi: 10.1177/14614448221102900

[ref59] Stevens N. Westerhof G. J. (2006). Partners and others: social provisions and loneliness among married Dutch men and women in the second half of life. J. Soc. Pers. Relat. 23, 921–941. doi: 10.1177/0265407506070474

[ref60] Su B.-C. Wu L.-W. Wu J.-P. (2023). Exploring the characteristics of YouTubers and their influence on viewers’ purchase intention: a viewers’ Pseudo-social interaction perspective. Sustainability 15:550. doi: 10.3390/su15010550

[ref61] Tidwell N. D. Eastwick P. W. Finkel E. J. (2013). Perceived, not actual, similarity predicts initial attraction in a live romantic context: evidence from the speed-dating paradigm. Pers. Relat. 20, 199–215. doi: 10.1111/j.1475-6811.2012.01405.x

[ref63] Turner J. R. (1993). Interpersonal and psychological predictors of parasocial interaction with different television performers. Commun. Q. 41, 443–453. doi: 10.1080/01463379309369904

[ref64] van Zalk M. Denissen J. J. A. (2015). Idiosyncratic versus social consensus approaches to personality: self-view, perceived, and peer-view similarity. J. Pers. Soc. Psychol. 109, 121–141. doi: 10.1037/pspp0000035, PMID: 25938702

[ref65] Vorderer P. Knobloch S. (1996). Parasoziale Beziehungen zu Serienfiguren. Ergänzung oder Ersatz? [Parasocial relationships with series characters. Complement or substitute?]. J. Media Psychol. 8, 201–216.

[ref66] Wang Q. Fink E. L. Cai D. A. (2008). Loneliness, gender, and parasocial interaction: a uses and gratifications approach. Commun. Q. 56, 87–109. doi: 10.1080/01463370701839057

[ref67] Webster G. D. Campbell J. T. (2022). Personality perception in game of thrones: character consensus and assumed similarity. Psychol. Pop. Media 12, 207–218. doi: 10.1037/ppm0000398

[ref68] Wong N. C. H. Lookadoo K. L. Nisbett G. S. (2017). “I’m Demi and I have bipolar disorder”: effect of parasocial contact on reducing stigma toward people with bipolar disorder. Commun. Stud. 68, 314–333. doi: 10.1080/10510974.2017.1331928

[ref69] Wortman J. Wood D. Furr R. M. Fanciullo J. Harms P. D. (2014). The relations between actual similarity and experienced similarity. J. Res. Pers. 49, 31–46. doi: 10.1016/j.jrp.2014.01.002

[ref70] Ye B. H. Fong L. H. N. Luo J. M. (2021). Parasocial interaction on tourism companies’ social media sites: antecedents and consequences. Curr. Issues Tour. 24, 1093–1108. doi: 10.1080/13683500.2020.1764915

